# The use of high-velocity, low-amplitude techniques in paediatric osteopathic care: two multinational cross-sectional surveys on clinical practice and educational curricula

**DOI:** 10.1186/s12998-026-00670-y

**Published:** 2026-08-10

**Authors:** Patrick L. S. van Dun, Lucas Bohlen, Julie Ellwood, Ana Paula A. Ferreira, Amie Steel, Jerry Draper-Rodi, Paul Vaucher

**Affiliations:** 1 Research Committee, Osteopathy Europe, Brussels, Belgium; 2 Clinical-Human Research Department, Foundation COME Collaboration, Pescara, Italy; 3 Research Department, Osteopathie Schule Deutschland, Hamburg, Germany; 4 Institute for ClinicalPsychology and Psychotherapy & Institute of Interdisciplinary Exercise Science and Sports Medicine, Medical School Hamburg, Hamburg, Germany; 5 UCO School of Osteopathy, Health Sciences University, Bournemouth, UK; 6 Centre for Osteopathic Research and Leadership,UCO School of Osteopathy, Health Sciences University, Bournemouth, UK; 7 Instituto Brasileiro de Osteopatia, Rio de Janeiro, Brazil; 8 School of Public Health, Faculty of Health, University of Technology, Sydney, Australia; 9 National Centre for Naturopathic Medicine, Faculty of Health, Southern Cross University, Lismore, Australia; 10 National Council for Osteopathic Research, Health Sciences University, London, UK; 11 University of Applied Sciences and Arts Western Switzerland (HES-SO), Delémont, Switzerland

**Keywords:** Manipulation, Osteopathic, Infant, Child, Adolescent, Cross-sectional studies

## Abstract

**Background:**

Up to one osteopathic consultation out of four involve infants and children. Although the clinical effectiveness of osteopathic care in paediatric populations remains inconclusive, available evidence suggests that it is generally safe. In contrast, high velocity, low amplitude technique (HVLA) has been associated with potential adverse effects. To inform a professional position on the use of HVLA techniques in paediatric care, this study investigated (1) the use of HVLA techniques by osteopathic practitioners in the treatment of infants, children, and adolescents, and (2) the extent to which HVLA techniques are included in osteopathic training programmes for these paediatric age groups.

**Methods:**

Two online cross-sectional surveys were conducted. The first targeted osteopathic practitioners and consisted of seven close-ended questions. The second survey was directed at osteopathic education institutes (OEIs) in Europe, Australia, Brazil, Israel, and New Zealand and consisted of three close-ended questions. Surveys were implemented via REDCap and made available in 11 languages. Participants were recruited between October 2025 and January 2026.

**Results:**

Among responding practitioners (n = 2607, response rate: 10.7%), 6.5% reported using HVLA in newborns or infants, 43.5% in children, and 78.3% in adolescents. Most respondents (54%) who apply HVLA to newborns and infants reported doing so “rarely”. Of the 62 responding OEIs (response rate: 30.7%), 91.4% offered osteopathic paediatric education; however, only 4.8% reported teaching HVLA for newborns and infants, 11.3% for children, and 56.4% for adolescents. Most respondents (70.2%) held an HVLA-opposed stance. Among the distinct practitioner profiles identified in the prioritisation of conditions for HVLA use in newborns and infants, the precaution-informed stance (11.2%) was the most strongly associated with paediatric practice and with the highest reported HVLA use in this age group.

**Conclusions:**

Most respondents did not use HVLA in newborns and infants, with utilisation increasing in older paediatric age groups, and the majority expressed opposition to paediatric HVLA. More permissive stances were associated with greater HVLA use and more training. The generalisability of the findings is limited by potential coverage and self-selection bias, possible duplicate responses, and the low and variable response rate to the survey of osteopathic practitioners. Given its continued application, an evidence-based position statement and robust research on the safety and effectiveness of HVLA in paediatrics are urgently needed.

**Supplementary Information:**

The online version contains supplementary material available at 10.1186/s12998-026-00670-y.

## Background

Osteopathy is a person-centred healthcare discipline designed to restore physical function and enhance well-being [1]. It utilises clinical reasoning to synthesise patients' lived experiences, diagnostic history, and physical examination [2]. Osteopathic care integrates hands-on manual therapy with patient education, lifestyle and exercise advice, and cognitive and behavioural support, evaluating the patient through a modern biopsychosocial lens rather than relying solely on traditional biomechanical frameworks [3]. Patients predominantly consult osteopathic practitioners for musculoskeletal complaints [4], and osteopathic treatment has been shown to be both safe and effective for conditions such as low back and neck pain [5–8]. Nonetheless, osteopathic practitioners also manage a broader range of patient populations and clinical conditions [9–28]. However, current systematic reviews and meta-analyses indicate that the available evidence for conditions like primary headaches, irritable bowel syndrome, and paediatric disorders remains limited and inconclusive, warranting additional high-quality research [5, 8]. Depending on the country, children under 2 years of age account for approximately 10–19.5% of patients receiving osteopathic care annually [29]. Thus, children—particularly infants—constitute a substantial proportion of patients receiving osteopathic care. The most common paediatric conditions in osteopathic practice include infantile postural asymmetry, plagiocephaly, excessive crying, infantile colic, and feeding and sleep disorders [30, 31].

To date, the evidence on the effectiveness of osteopathic care for paediatric conditions remains mixed. Meta-analyses have reported significant reductions in (1) crying time in infants [15], particularly in those with infantile colic [20, 32], and (2) both the cost [22] and duration [20, 22] of hospitalisation in preterm infants, although contradictory findings also exist [24]. Conversely, osteopathic care has not been shown to significantly improve infantile postural asymmetry, sleep in infants with colic, or gross motor function in children with cerebral palsy [20]. However, many reviews emphasise the limited quality and quantity of primary studies, which impedes the feasibility of meta-analyses for several paediatric conditions and underscores the need for high-quality clinical trials to draw more definitive conclusions [15, 20, 24, 32–34]. Importantly, no serious adverse events (AEs) were reported across these reviews [15, 20, 22, 24, 32–34]. However, systematic reviews [15, 20, 22, 24, 32, 34] of osteopathic paediatric care reveal that 20–97% of identified clinical trials fail to report the presence or absence of adverse events, revealing important methodological and reporting issues.

Osteopathic practitioners employ a wide range of manual techniques in patient care [35, 36], while high velocity, low amplitude (HVLA) techniques rank among the most frequently used worldwide (~ 34–85%) [4]. Recognising that terminology varies across manual therapy disciplines and literature, it is reasonable to infer that terms such as 'HVLA' and 'spinal manipulation' share significant conceptual and clinical overlap [35]. Within this study, they are treated as functionally analogous modalities. For this study, the research team used the following definition:

HVLA: “High velocity, low amplitude also called thrust treatment method. An osteopathic method in which the restrictive barrier is engaged in one or more planes of motion and then a rapid, therapeutic force of brief duration traveling a short distance is applied within the anatomic range of motion.” [36]

However, evidence suggests a clear preference for non-HVLA techniques in infants and children [37], with balanced membranous/ligamentous tension, fluid techniques, myofascial release, and soft tissue techniques being most commonly employed [30, 38]. In turn, findings from a single osteopathic clinic in the United States indicate that HVLA techniques are rarely used, and that its use increases with patient age, from less than 1% in children under two years of age to more than 10% in adolescents aged 12–21 years [30].

Regarding the effectiveness of HVLA techniques, systematic reviews have either found no evidence supporting their use for any paediatric condition [39] or have been unable to draw conclusions due to the low methodological quality of the available studies [40]. Conversely, several reviews have documented predominantly mild AEs following spinal manipulations in paediatric patients, with moderate, serious, and indirect AEs reported much less frequently [40–43]. Although some serious AEs may have been related to pre-existing underlying pathologies rather than the intervention itself [40, 42]. Another review concluded that it remains unclear whether spinal manipulative therapy increases the risk for AEs in children under ten years of age, noting that reported AEs were predominantly mild (e.g., crying) and rarely serious (e.g., rib fracture) [44]. Nevertheless, AE reporting is inconsistent and sparse across the literature, and further research is required to establish the safety profile of spinal manipulations in paediatric populations [39–41, 44, 45]. Taken together, current evidence suggests that, for paediatric patients, the potential risks of spinal manipulation may outweigh the uncertain benefits [39, 46].

Against this backdrop, the application of HVLA techniques in paediatric populations has come under increasing scrutiny from professional bodies, governmental agencies, and research task forces. The controversy initially sparked in Australia, where the circulation of a video showing a chiropractor performing cervical manipulation on an infant elicited substantial public and media concern [45], which in turn prompted a governmental investigation into this practice [46]. Due to insufficient evidence of clinical effectiveness and persistent uncertainty regarding the potential for harm, the government subsequently recommended that manual therapists refrain from performing spinal manipulation in children younger than 12 years [46]. Following this advisory, Australian chiropractic, physiotherapy, and osteopathic professional associations issued position statements and interim policies that endorsed and echoed these governmental recommendations [47–49].

Internationally, a physiotherapy-led research task force conducted a series of literature reviews and expert surveys [39, 50–52] to inform a position statement on the use of spinal manipulation in paediatric populations [45, 53]. This statement recommended against the use of (1) spinal manipulation in infants, (2) cervical and lumbar manipulation in children, and (3) spinal manipulation for non-musculoskeletal conditions in infants, children, and adolescents [45]. In contrast, a clinical practice guideline for chiropractors emphasised the need to modify manipulative techniques to reflect the developmental stage of paediatric patients, but did not explicitly discourage the use of spinal manipulation in this population [54]. These divergent recommendations stem from the same core limitation: the sparse and methodologically weak evidence base regarding both the effectiveness and safety of spinal manipulations in paediatric populations—a constraint acknowledged across professional groups but balanced differently alongside clinical expertise and patient values when developing recommendations [52, 54].

The osteopathic profession has not yet issued a position statement regarding the use of HVLA techniques in paediatric populations. Nonetheless, a Dutch clinical guideline [55] and a non–peer-reviewed statement issued by an Australian osteopathic professional association [49] recommended against the use of spinal manipulation in infants and in children under 2 and 12 years of age, respectively, citing insufficient evidence of clinical benefit and substantial public and regulatory concerns surrounding this practice. However, to date, there is insufficient evidence describing whether osteopathic practitioners do or do not use HVLA techniques in paediatric populations. This lack of empirical data complicates the development of a well-informed, evidence-based position statement for the osteopathic profession.

Accordingly, the objectives of this study are twofold: (1) to examine the extent to which osteopathic practitioners use HVLA techniques when treating infants (< 2 years), children (2–12 years), and adolescents (13–17 years); and (2) to determine whether HVLA techniques are included in osteopathic training programmes for these paediatric age groups.

## Methods

Two online cross-sectional surveys, developed within the research team, were designed to address the research questions. The protocol, as submitted to the ethical board prior to data collection, is made available on Zenodo (1-Protocol-HVLAPed-250410.pdf; https://zenodo.org/records/19097090). The reporting guideline utilised was the Consensus-Based Checklist for Reporting of Survey Studies (CROSS) [56] (Additional file 1).

### Practitioner survey

The first survey targeted osteopathic practitioners who were members of the current 27 osteopathic professional associations affiliated with the European umbrella organisation Osteopathy Europe (OE), in addition to the Swiss Osteopathic Association, Osteopathy Australia and Osteopaths New Zealand (Table 1).

**Table 1:** Number of osteopathic members of the included professional organisations per country

Country	Professional Association	N	RR	%
Australia	Osteopathy Australia	2478	36	1.5
Austria	Austrian Association of Osteopathy	529	21	4.0
Belgium	Osteopathy Belgium	894	124	13.9
Brazil	Brazilian Registry of Osteopaths	183	48	26.2
Cyprus	Cyprus Osteopathic Association	15	2	13.3
Denmark	Danish Association of Osteopaths	312	69	22.1
Estonia	Baltic Osteopathic Association	18	2	11.1
Finland	Finnish Osteopathic Association	120	56	46.7
France	French Osteopathic Association French Union of Osteopaths	1732	477	27.5
Germany	Association of Osteopaths Germany	5340	503	9.4
Greece	Panhellenic Osteopathic Association	65	5	7.7
Iceland	Icelandic Osteopathic Association	4	0	0.0
Ireland	Osteopathic Council of Ireland	177	95	53.7
Israel	Israeli Register of Osteopaths	200	8	4.0
Italy	Italian Register of Osteopaths	5220	473	9.1
Lithuania	Lithuanian Osteopathic Association	14	3	21.4
Luxembourg	Luxemburg Association of Osteopaths	92	24	26.1
Malta	Malta Association of Osteopaths	10	3	30.0
Netherlands	Netherlands Register for Osteopathy Dutch Professional Association for Osteopathy	633	128	20.2
New Zealand	Osteopaths New Zealand	346	75	21.7
Norway	Norwegian Association of Osteopaths	401	71	17.7
Poland	Towarzystwo Osteopatow Polskich	19	3	15.8
Portugal	Associação dos Osteopatas de Portugal	207	34	16.4
Slovenia	Slovenian Association of Osteopaths	4	0	0.0
Spain	Spanish Osteopathic Association	438	70	16.0
Sweden	Swedish Osteopathic Association	244	36	14.8
Switzerland	Swiss Osteopathic Association	966	63	6.5
United Kingdom	Institute of Osteopathy	3600	160	4.4
Other*			14	
Unknown			3	
Total		24,261	2607	10.7

N, Total number of invited osteopathic practitioners; RR, Response Rate. * ‘Other’ refers to countries outside the 28 countries from which osteopathic practitioners were invited to participate in this survey

Associations invited their members through email and social media. The survey assessed the use of HVLA techniques across paediatric age groups. The questionnaire included seven mandatory close-ended items. Frequency of HVLA use was investigated using five step Likert scales ranging from “never” to “very often”. All professional associations were informed of the study and asked to distribute the survey to their members. In total, 24,261 osteopathic practitioners from 28 countries were invited. An e-campaign containing distributable promotional material was provided to facilitate recruitment through social media, websites, and newsletters.

### OEI/CPD provider survey

The second survey targeted Osteopathic Education Institutes (OEIs) and Continuing Professional Development (CPD) providers in 24 European countries, Australia, Brazil, Israel and New Zealand (Table 1). It collected information on paediatric curricula and whether HVLA techniques are taught for treating infants. This questionnaire consists of three close-ended items. The list of OEIs and CPD providers was compiled by representatives of OE member organisations and by collaborators in Australia, New Zealand and Switzerland. In total, 212 institutes and providers were invited.

### Translation process

Both questionnaires and the Participant Information Sheets (PIS) were translated from English to Danish, Dutch, Finnish, French, German, Greek, Italian, Portuguese, Spanish, and Swedish, using DeepL (3a–3b; https://zenodo.org/records/19097090). Native speakers affiliated with OE member organisations reviewed and adapted each version before back-translation with DeepL. The process followed WHO recommendations for forward–backward translation [57, 58].

### Pilot testing

Two pilot studies were conducted to support questionnaire validation. The first assessed content and face validity among members of the OE Research Committee (n = 12), representing 12 of the 25 member countries, and two representatives from both the Australian and New Zealand associations (N = 16). Revisions were implemented based on their feedback.

The second pilot assessed linguistic clarity. Two native-speaking osteopathic practitioners for each language version reviewed wording and phrasing (N = 22), resulting in further refinements.

### Data protection and ethics

In accordance with Directive 2018/1725CE of the European Parliament [59], all data were anonymised and handled via REDCap, a secure platform for online surveys and research databases [60, 61]. REDCap was hosted by the HES-SO on a secured server at the School of Engineering and Architecture in Fribourg. No sensitive information was collected, and IP addresses were not accessible to researchers.

Ethics approval was granted by the Institutional Review Board of the Non-profit Foundation COME Collaboration, the Brazilian Centro Universitario Augusto Motta (UNISUAM), and the University of Technology Sydney (2a–2c; https://zenodo.org/records/19097090).

### Recruitment and data collection

Recruitment was planned for a three-month period. Professional associations were free to send reminders at their discretion and were informed twice monthly about the response rate. REDCap automatically issued reminders to OEIs and CPD providers in case of non-response. Invitation emails contained a link to the PIS, followed by access to the survey. Participation was voluntary. REDCap alerted respondents to missing items to minimise incomplete entries. Explicit consent was obtained via a dedicated checkbox before survey initiation. Unique visitor metrics could not be implemented in REDCap because professional associations were prohibited from sharing member contact details under EU data protection regulations and declined to distribute individual-specific access links. However, associations provided the total number of invited members, enabling the estimation of country-specific response rates. Post-stratification weighting or propensity score adjustments were not performed, as the aggregate demographic characteristics of the associations' membership were unavailable or logistically unfeasible to collect, leaving the true target population parameters unknown.

### Data cleaning

Only responses from osteopathic practitioners who provided their consent and those having completed the survey to the end were included. Potential duplicates could not be identified and were therefore not removed if present. A conservative approach was used to address incoherences in responses. If the total percentage of patients consulted monthly by age group was not 100%, the percentage allocated to adults was changed to meet that expectation. Some participants (n = 12) reported using cervical HVLA techniques whilst reporting never using HVLA in general. This inconsistency was treated as them reporting using cervical HVLA techniques correctly. When respondents indicated that no circumstances could justify the use of HVLA techniques, any additional circumstances they reported were disregarded, as these would contradict their initial statement. Those that reported no conditions at all were considered to think that no conditions could justify the use of HVLA techniques.

### Sample size estimate

With a 95% confidence level, a target population of 25,000 osteopaths, and a 2% margin of error, the required sample size to estimate the prevalence of osteopathic practitioners using HVLA techniques in paediatrics was 2191 (equivalent to a response rate of 8%).

### Statistical analysis

Descriptive statistics were used to report the prevalence of HVLA use and teaching among practitioners and OEIs. Prevalence estimates were calculated using the exact method with a 95% confidence level. HVLA use in infants was dichotomised as “none” versus “rarely” to “very often”. Country-stratified analyses were conducted for countries with ≥ 50 respondents. Logistic regression was used to identify associated factors. Univariate analysis was first run setting *p*-value threshold at *p* ≤ 0.1. Only these factors were maintained in multivariate analysis using a backward step approach removing most non-significant factors until all remaining factors were significant (*p* < 0.05). No adjustment was done for multiple testing. Reference groups were defined as those with the largest number of responders. Survey estimates are reported with 95% confidence intervals. Distinct practitioner profiles in the prioritisation of conditions for HVLA use in newborns and infants were identified using latent class analysis with maximum likelihood estimation. Competing models specifying two to seven latent classes were evaluated. Model fit was primarily assessed using the Bayesian Information Criterion (BIC), with the Akaike Information Criterion (AIC) used as a secondary indicator in cases of BIC instability. Final model selection was based on the lowest BIC in conjunction with the conceptual coherence and clinical interpretability of the resulting class structure [62]. All analyses were done using Stata StataCorp. 2017 (*Stata Statistical Software: Release 15*. College Station, TX: StataCorp LLC). The datasets supporting the conclusions of this article are available on Zenodo, [https://zenodo.org/records/19097090]. Details on commands and full output file are also made available on Zenodo (4–5).

## Results

### Description of respondents

The surveys ran for 92 days from 5 October 2025 to 5 January 2026, and 2607 osteopathic practitioners from 28 invited countries gave their consent and completed the online questionnaire to the end; 196 entries were not accounted for, 53 for not giving their consent, 143 for not completing the questionnaire to the end. Estimated response rates varied considerably between countries with a median of 10.7%, and ranging from 0.0% for Iceland and Slovenia to 53.7% for respondents from Ireland. Details on country-by-country response rate and participation is provided in Table 1. Compared to the other nationalities, Danish and Finnish respondents tended to have less years of working experience, and UK respondents more. Table 2 describes respondents from countries with 50 or more respondents. One patient in 7 seen by respondents is estimated to be a newborn or infant (age < 2 years).

**Table 2:** Description of responding osteopathic practitioners

	All n = 2607	B n = 124	DK n = 69	FIN n = 56	F n = 477	D n = 503	IRL n = 95	I n = 473	NL n = 128	NZ n = 75	N n = 71	E n = 70	CH n = 63	UK n = 160	Other n = 243
*Duration of working experience as an osteopath; n (%)*															
< 2 years	151 (5.8)	5 (4.0)	10 (14.5)	6 (10.7)	33 (6.9)	24 (4.8)	7 (7.4)	25 (5.3)	6 (4.7)	4 (5.3)	4 (5.6)	1 (1.4)	5 (7.9)	9 (4.9)	12 (4.9)
2 – 5 years	434 (16.6)	11 (8.9)	21 (30.4)	20 (35.7)	63 (13.2)	82 (16.3)	18 (18.9)	109 (23.0)	18 (14.1)	9 (12.0)	12 (16.9)	1 (1.4)	14 (22.2)	11 (6.9)	45 (18.5)
6 – 10 years	528 (20.3)	18 (14.5)	16 (23.2)	17 (30.4)	74 (15.5)	109 (21.7)	13 (13.7)	134 (28.3)	18 (14.1)	8 (10.7)	20 (28.2)	15 (21.4)	5 (7.9)	19 (11.9)	62 (25.5)
11 – 20 years	871 (33.4)	46 (37.1)	18 (26.1)	9 (16.1)	222 (46.5)	158 (31.4)	21 (22.1)	143 (30.2)	48 (37.5)	23 (30.7)	23 (32.4)	30 (42.9)	18 (28.6)	42 (26.2)	70 (28.8)
21 – 30 years	438 (16.8)	24 (19.3)	2 (2.9)	3 (5.4)	54 (11.3)	112 (22.3)	28 (29.5)	44 (9.3)	28 (21.9)	13 (17.3)	11 (15.5)	22 (31.4)	19 (30.2)	35 (21.9)	43 (17.7)
31 – 40 years	129 (4.9)	9 (7.3)	2 (2.9)	1 (1.8)	20 (4.2)	13 (2.6)	7 (7.4)	14 (2.0)	8 (6.2)	8 (10.7)	1 (1.4)	1 (1.4)	2 (3.2)	33 (20.6)	10 (4.1)
> 40 years	56 (0.4)	11 (8.9)	0 (0)	0 (0)	11 (2.3)	5 (1.0)	1 (1.1)	4 (0.8)	2 (1.6)	10 (13.3)	0 (0)	0 (0)	0 (0)	11 (6.9)	1 (0.4)
*Estimated % of patients by age group;* $\bar{x}$*(SD)*															
Newborns and infants (< 2 years)	13.6 (16.2)	17.4 (18.5)	15.1 (18.3)	11.0 (12.5)	11.8 (12.9)	13.8 (14.0)	15.0 (17.8)	14.1 (17.2)	18.4 (14.0)	8.3 (10.3)	7.6 (10.6)	11.7 (14.5)	13.4 (15.0)	10.0 (15.8)	16.6 (23.8)
Children (2 – 12 years)	8.2 (7.0)	9.0 (6.1)	4.7 (3.3)	7.2 (6.7)	9.0 (6.2)	9.5 (7.6)	7.7 (6.7)	8.7 (7.0)	6.6 (5.5)	6.2 (5.6)	4.3 (5.2)	10.5 (10.5)	10.6 (8.1)	3.7 (5.3)	7.5 (7.3)
Adolescents (13 – 17 years)	9.7 (8.0)	10.0 (6.9)	7.1 (6.0)	6.7 (5.9)	10.6 (6.3)	10.6 (10.3)	9.6 (7.0)	12.0 (9.1)	6.7 (5.1)	7.8 (5.3)	8.5 (7.9)	10.0 (7.0)	10.5 (5.3)	5.1 (5.4)	8.4 (7.3)
Adults (≥ 18 years)	68.6 (21.8)	63.7 (21.2)	73.2 (18.9)	75.1 (17.6)	68.6 (18.4)	66.1 (22.0)	68.1 (22.3)	65.1 (22.0)	68.3 (17.9)	77.7 (16.7)	79.7 (16.8)	67.8 (24.6)	65.5 (22.7)	81.2 (20.7)	67.7 (27.3)

B, Belgium; DK, Denmark; FIN, Finland; F, France; D, Germany; IRL, Ireland; I, Italy; NL, Netherlands; NZ, New Zealand; N, Norway; E, Spain; CH, Switzerland; UK, United Kingdom

Sixty-five osteopathic education institutions contributed, resulting in a 30.7% response rate. One stated their institution did not want to contribute, two stopped before answering all three questions, leaving responses from 62 OEIs.

### Use of HVLA techniques for newborns and infants (< 2 years)

**Fig. 1:**
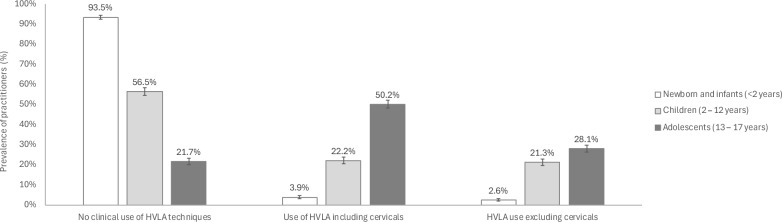
Observed reported prevalence of HVLA use by osteopathic practitioners on different age groups. Error bars correspond to 95%CIs

The overall prevalence of responding practitioners reporting the use of HVLA techniques on newborns or infants was 6.5% [CI95% 5.6–7.5]; and for cervical HVLA, it was 3.9% [CI95% 3.2–4.7] (Fig. 1).

The use of HVLA techniques on the cervical spine for paediatric care varied substantially across countries (Table 3). Respondents from Finland, the Netherlands, and the UK seldom report using them (< 1%), whereas respondents working in Germany (14.1%), Spain (11.4%), or Switzerland (9.5%) report much more frequent usage. Six respondents (0.23%) reported using HVLA techniques on newborn infants “very often”. Sixty-seven respondents (2.6%) reported using HVLA techniques but not using cervical HVLA techniques. Among those using cervical HVLA techniques on newborns and infants (n = 102), 55 (53.9%) use such techniques”rarely”.

**Table 3:** Prevalence of osteopathic practitioners using HVLA techniques as part of paediatric patient management

	All n = 2607	B n = 124	DK n = 69	FIN n = 56	F n = 477	D n = 503	IRL n = 95	I n = 473	NL n = 128	NZ n = 75	N n = 71	E n = 70	CH n = 63	UK n = 160	Other n = 243
*Newborn and infants (< 2 years)*															
No clinical use of HVLA	2438 (93.5%)	120 (96.8%)	64 (92.8%)	56 (100%)	457 (95.8%)	432 (85.9%)	90 (94.7%)	450 (95.1%)	128 (100%)	73 (97.3%)	67 (94.4%)	62 (88.6%)	57 (90.5%)	159 (99.4%)	223 (91.8%)
Clinical use of HVLA including cervicals	102 (3.9%)	1 (0.8%)	2 (2.9%)	0 (0%)	16 (3.4%)	43 (8.6%)	3 (3.2%)	13 (2.7%)	0 (0%)	1 (1.3%)	1 (1.4%)	7 (10.0%)	6 (9.5%)	0 (0%)	9 (3.7%)
Clinical use of HVLA excluding cervicals	67 (2.6%)	3 (2.4%)	3 (4.3%)	0 (0%)	4 (0.8%)	28 (5.6%)	2 (2.1%)	10 (2.1%)	0 (%)	1 (1.3%)	3 (4.2%)	1 (1.4%)	0 (0%)	1 (0.6%)	11 (4.5%)
*Children (2–12 years)*															
No clinical use of HVLA	1473 (56.5%)	59 (47.6%)	40 (58.0%)	53 (94.6%)	258 (54.1%)	211 (41.9%)	57 (60.0%)	288 (60.9%)	93 (72.7%)	42 (56.0%)	50 (70.4%)	33 (47.1%)	30 (47.6%)	115 (71.9%)	144 (59.3%)
Clinical use of HVLA including cervicals	579 (22.2%)	32 (25.8%)	19 (27.6%)	1 (1.8%)	76 (15.9%)	164 (32.6%)	16 (16.8%)	104 (22.0%)	13 (10.2%)	18 (24.0%)	15 (21.1%)	25 (35.7%)	20 (31.7%)	21 (13.1%)	55 (22.6%)
Clinical use of HVLA excluding cervicals	555 (21.3%)	33 (26.6%)	10 (14.5%)	2 (3.6%)	143 (30.0%)	128 (25.5%)	22 (23.2%)	81 (17.1%)	22 (17.2%)	15 (20.0%)	6 (8.5%)	12 (17.1%)	13 (20.6%)	24 (15.0%)	44 (18.1%)
*Adolescents (13–17 years)*															
No clinical use of HVLA	565 (21.7%)	17 (13.7%)	18 (26.1%)	38 (67.9%)	81 (17.0%)	97 (19.3%)	17 (17.9%)	102 (21.6%)	39 (30.5%)	12 (16.0%)	16 (22.5%)	13 (18.6%)	10 (15.9%)	41 (25.6%)	64 (26.3%)
Clinical use of HVLA including cervicals	1310 (50.2%)	81 (65.3%)	37 (53.6%)	6 (10.7%)	177 (37.1%)	271 (53.9%)	42 (44.2%)	285 (60.3%)	40 (40.2%)	48 (64.0%)	42 (59.2%)	45 (64.3%)	35 (55.6%)	72 (45.0%)	129 (53.1%)
Clinical use of HVLA excluding cervicals	732 (28.1%)	26 (21.0%)	14 (20.3%)	12 (21.4%)	219 (45.9%)	135 (26.8%)	36 (37.9%)	86 (18.2%)	49 (38.3%)	15 (20.0%)	13 (18.3%)	12 (17.1%)	18 (28.6%)	47 (29.4%)	50 (20.6%)

B, Belgium; DK, Denmark; FIN, Finland; F, France; D, Germany; IRL, Ireland; I, Italy; NL, Netherlands; NZ, New Zealand; N, Norway; E, Spain; CH, Switzerland; UK, United Kingdom

### Use of HVLA techniques for children (2–12 years)

Nearly half of respondents (43.5% [CI95% 41.6– 45.4]) use HVLA techniques on children, and little over a fifth (22.2% [CI95% 20.6–23.9]) use cervical HVLA techniques on children (Fig. 1). One hundred percent of practitioners who use HVLA “very often” also use cervical HVLA in children, compared to 36.2% of practitioners who 'rarely' use HVLA. Germany, Spain, and Switzerland were the only countries with 30% or more respondents reporting the use of cervical HVLA; and Finland the only one below 10% (Table 3).

### Use of HVLA techniques for adolescents (13–17 years)

A majority of respondents reported using HVLA in adolescents (78.3% [CI95% 76.7–79.9]), and approximately half reported using cervical HVLA techniques (50.2% [CI95% 48.3–52.2]) (Fig. 1). Most respondents (124/136; 91.2%) using HVLA ‘very often’ also used cervical HVLA, compared to 37.7% (232/384) of those using them ‘rarely’. France was the country in which most respondents used HVLA on adolescents but not on the cervical spine (219/477; 45.9%).

### Education and HVLA technique

Of the 62 responding OEIs, 57 (91.4%) reported providing education in paediatric osteopathic care: 50 (80.6%) for newborns and infants, 51 (82.3%) for children, and 54 (87.1%) for adolescents. Thirty-eight OEIs (61.3%) included these in undergraduate programmes, and 32 (51.6%) in postgraduate programmes. Nineteen (30.6%) of OEIs provided paediatric training solely for postgraduate education. Three institutions reported teaching HVLA techniques for newborns and infants (4.8%), 7 (11.3%) for children, and 35 (56.4%) for adolescents. This is taught in undergraduate programmes by 2 OEIs for newborns and infants, 6 for children, and 25 for adolescents. Institutions that teach cervical HVLA techniques (n = 3; 4.8%) for newborns and infants are the same as those teaching HVLA techniques in general. Some OEIs do not teach HVLA techniques on cervicals but do for other regions; 1 for children, and 10 for adolescents.

Table 4 reports the type of training followed by responding practitioners on HVLA use in paediatric osteopathic care.

**Table 4:** Reported received education for HVLA techniques as part of paediatric patient management

Newborns and infants (age < 2 years)	All	No clinical use of HVLA	Clinical use of HVLA	
			Including cervicals	Excluding cervicals
	n = 2607	n = 2438	n = 102	n = 67
Type of training				
No training	1971 (75.6%)	1941 (79.6%)	6 (5.9%)	24 (35.8%)
Undergraduate (Type I or II)	297 (11.4%)	230 (9.4%)	48 (47.1%)	19 (28.4%)
Postgraduate (University postgraduate degree or CPD)	339 (13.0%)	267 (11.0%)	48 (47.1%)	24 (35.8%)
Practical supervision	251 (9.6%)	167 (6.8%)	59 (57.8%)	25 (37.3%)

Children (age 2 – 12 years)	n = 2607	n = 1473	n = 579	n = 555
Type of training				
No training	1362 (52.2%)	1108 (75.2%)	63 (10.9%)	191 (34.4%)
Undergraduate (Type I or II)	728 (27.9%)	218 (14.8%)	298 (51.5%)	212 (38.2%)
Postgraduate (University postgraduate degree or CPD)	517 (19.8%)	147 (10.0%)	218 (37.6%)	152 (27.4%)
Practical supervision	656 (25.2%)	157 (10.7%)	309 (53.4%)	190 (34.2%)

Adolescents (age 13 – 17 years)	n = 2607	n = 565	n = 1310	n = 732
Type of training				
No training	601 (23.0%)	324 (57.4%)	105 (8.0%)	172 (23.5%)
Undergraduate (Type I or II)	1342 (51.5%)	180 (31.9%)	781 (59.6%)	381 (52.1%)
Postgraduate (University postgraduate degree or CPD)	664 (25.5%)	61 (10.8%)	424 (32.4%)	179 (24.4%)
Practical supervision	1273 (48.8%)	131 (23.2%)	812 (62.0%)	330 (45.1%)

CPD, Continuous Professional Development; HVLA, High Velocity Low Amplitude techniques

### Practitioners’ stances towards conditional use of HVLA techniques in newborn and infants

A total of 1830 (70.2%) respondents considered that no condition whatsoever could justify the use of HVLA techniques in newborns and infants (i.e. HVLA-opposed stance). From the 777 respondents who considered that HVLA techniques could be used on newborns and infants under certain conditions, 75.0% selected ‘sufficient training’, 65.4% ‘parent consent’, 65.2% ‘absence of anatomical malformations’, 56.2% ‘benefits outweighs risks’, 52.4% ‘failure of other osteopathic manipulative techniques’, 49.2% ‘evidence of mechanisms’, and 32.7% ‘patient consent’. In addition to these seven proposed possible conditions, respondents also suggested practitioners preferences, level of confidence, and ability to self-reflect and be aware of one's own limits.

**Fig. 2:**
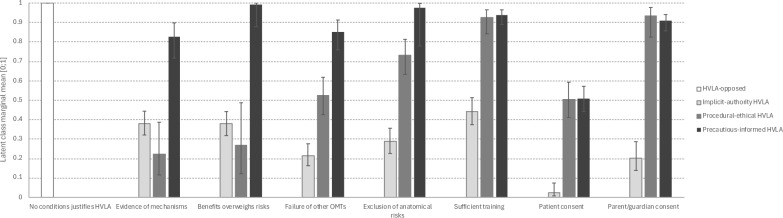
Identified stances for using HVLA on newborns and infants (N = 2607). The Latent class marginal means (LCMM) correspond to the weight of each condition for each identified stance. Error bars correspond to 95%CI for LCM

Latent Class Analysis was then used to distinguish permissive stances, defined as distinct groupings of conditions (Table 5). From 777 respondents who provided conditions, three distinct stances were identified: precaution-informed stance (n = 292; 11.2%), implicit-authority stance (n = 285; 10.9%), and procedural-ethical stance (n = 200; 7.7%) (Fig. 2). Descriptions of practitioners by justificatory stance regarding HVLA use in newborns and infants are reported in Table 6.

**Table 5:** Presentation of the different stances on the use of HVLA techniques for the paediatric population

Stance	Characterised by …
HVLA-opposed	the position that no clinical, ethical, or contextual conditions can justify the use of HVLA techniques in the paediatric population, leading to its categorical exclusion in this population
Implicit-authority HVLA	acceptance of HVLA use with minimal explicit constraints, where its legitimacy does not depend on clearly specified ethical requirements (such as consent), mechanistic evidence, or formal safety criteria, and is instead assumed through implicit professional authority rather than articulated justification
Procedural-ethical HVLA	the view that HVLA use may be justified when procedural and ethical conditions are met, notably adequate practitioner training and explicit parental or guardian consent, while placing limited emphasis on mechanistic explanation or formal benefit–risk evaluation
Precaution-informed HVLA	the view that HVLA use may be justified when informed by a precautionary appraisal, in which anticipated benefits are expected to outweigh risks and are considered alongside contextual safeguards such as exclusion of anatomical risks, practitioner training, and consent, reflecting an evidence-informed approach constrained by benefit–risk considerations

**Table 6:** Description of practitioners by justificatory stance regarding HVLA use in newborns and infants

	HVLA-opposed n = 1830 N (%)	Implicit-authority n = 285 N (%)	Procedural-ethical n = 200 N (%)	Precautious-informed n = 292 N (%)
*Use of HVLA*				
No clinical use of HVLA techniques	1801 (73.9%)	245 (10.0%)	164 (6.7%)	228 (9.4%)
Use of HVLA techniques on cervicals	9 (8.8%)	26 (25.5%)	23 (22.6%)	44 (43.1%)
HVLA use excluding cervicals	20 (29.8%)	14 (20.9%)	13 (19.4%)	20 (29.8%)
*Country*				
Belgium	98 (79.0%)	10 (8.1%)	4 (3.2%)	12 (9.7%)
Denmark	44 (63.8%)	8 (11.6%)	9 (13.0%)	8 (11.6%)
Finland	54 (96.4%)	1 (1.8%)	0 (0%)	1 (1.8%)
France	375 (78.6%)	47 (9.8%)	17 (3.6%)	38 (8.0%)
Germany	231 (45.9%)	82 (16.3%)	71 (14.1%)	119 (23.7%)
Ireland	78 (82.1%)	9 (9.5%)	3 (3.2%)	5 (5.3%)
Italy	329 (69.6%)	55 (11.6%)	44 (9.3%)	45 (9.5%)
Netherlands	112 (87.5%)	10 (7.8%)	2 (1.6%)	4 (3.1%)
New Zealand	59 (78.7%)	6 (8.0%)	2 (1.6%)	5 (6.7%)
Norway	49 (69.1%)	7 (9.9%)	12 (16.9%)	3 (4.2%)
Spain	45 (64.3%)	12 (17.1%)	6 (8.6%)	7 (10.0%)
Switzerland	36 (57.1%)	9 (14.3%)	6 (9.5%)	12 (19.1%)
United Kingdom	141 (88.1%)	3 (1.9%)	10 (6.2%)	6 (3.8%)
Other	179 (73.7%)	26 (10.7%)	11 (4.5%)	27 (11.1%)
*Education in HVLA for newborns and infants*				
Training				
None	1521 (77.2%)	174 (8.8%)	106 (5.4%)	170 (8.6%)
Undergraduate (Type I or II)	146 (49.2%)	52 (17.5%)	42 (14.1%)	57 (19.2%)
Postgraduate (*University postgraduate degree or* CPD)	163 (48.1%)	59 (17.4%)	52 (15.3%)	65 (19.2%)
Practical supervision	94 (37.5%)	56 (22.3%)	39 (15.5%)	62 (24.7%)
*Years of experience as an osteopath*				
≤ 10 years	751 (67.5%)	127 (11.4%)	96 (8.6%)	139 (12.5%)
11–20 years	622 (71.4%)	93 (10.7%)	56 (6.4%)	100 (11.5%)
21–30 years	307 (70.1%)	44 (10.0%)	41 (9.4%)	46 (10.5%)
> 30 years	150 (81.1%)	21 (11.3%)	7 (3.8%)	7 (3.8%)
*Proportion of newborns and infant in consultations*				
< 10%	862 (68.8%)	148 (11.8%)	101 (8.1%)	142 (11.3%)
10–49% ≥ 50%	880 (71.6%) 88 (70.4%)	130 (10.6%) 7 (5.6%)	93 (7.6%) 6 (4.8%)	126 (10.2%) 24 (19.2%)

Percentages are per row

Among those holding an HVLA-opposed stance, 98.4% (1801/1830) consistently reported not using HVLA techniques on newborns and infants themselves. The HVLA-opposed stance was characterised by limited exposure to HVLA training, with 83.1% reporting no training. Compared to other stances, practitioners with more than 30 years of experience were slightly more represented (8.2% vs. 7.4% / 3.5% / 2.4%). Despite their categorical opposition, HVLA-opposed respondents were not totally marginal to paediatric practice as 4.8% reported a newborn and infant caseloads ≥ 50%, which was similar to other stances (2.5% / 3.0% / 8.2%). This suggests that opposition to HVLA can coexists with substantial paediatric exposure.

Compared with respondents holding an HVLA-opposed stance, those in the permissive stances reported greater overall use of HVLA techniques (18.0% vs. 1.6%), particularly cervical HVLA (12.0% vs. 0.5%), and a higher proportion had received specific training in HVLA for newborns and infants (42.1% vs. 16.9%).

Quantitatively, the implicit-authority stance represents a weakly constrained permissive position, characterised by neither clear exclusion criteria nor a strong justificatory framework. The procedural-ethical stance emphasises authorisation through process (e.g., training and consent) rather than precautionary outcome evaluation or senior expertise. In contrast, the precautious-informed stance reflects active HVLA use within an explicit benefit–risk framework, supported by training and supervision and embedded in substantial paediatric clinical exposure (Fig. 2).

### Predictors of use of HVLA techniques in newborns and infants

Years of experience, formal training in the use of HVLA techniques in newborns and infants, having received practical supervision, and all permissive stances toward HVLA were all positively and independently associated with increased odds of HVLA use, including cervical HVLA techniques, after adjustment for covariates (R^2^ = 0.344 for any HVLA use; R^2^ = 0.439 for cervical HVLA). In contrast, the proportion of newborns and infants in the clinical caseload was not independently associated with HVLA use after adjustment. Overall, HVLA use was most strongly associated with adopting a precaution-informed stance (aOR = 11.5) and having had prior HVLA training (undergraduate aOR = 7.3; postgraduate aOR = 6.5; practical supervision aOR = 2.6). For detailed estimates, see Table 7.

**Table 7:** Use of HVLA on newborns and infants and association to training, experience, country of practice, and stances (N = 2,607)

	All HVLA		Cervical HVLA	
	Crude OR [CI95%]	Adjusted OR [CI95%]	Crude OR [CI95%]	Adjusted OR [CI95%]
Years of experience*	1.29 [1.1;1.5]	1.30 [1.11;1.53]	1.39 [1.2;1.6]	1.45 [1.2;1.8]
Proportion of patients^†^	1.11 [1.02;1.2]	n.s	1.13 [1.03;1.2]	n.s
*Training*				
*Type of training*				
None (N = 1971)	Ref	Ref	Ref	Ref
Undergraduate (Type I or II) (N = 297)	18.8 [12.0;29.6]	7.3 [4.3;12.4]	63.1 [26.7;149]	20.7 [8.2;52.2]
Postgraduate (*University postgraduate degree or* CPD) (N = 339)	17.4 [11.2;27.2]	6.5 [3.9;11.0]	54.0 [22.9;127.3]	18.3 [7.3;46.3]
Practical supervision (N = 251)	13.4 [9.6;18.9]	2.6 [1.7;3.9]	16.5 [10.9;25.1]	2.4 [1.4;3.9]
*Country of practice*				
Germany (N = 503)	2.8 [2.0;3.9]	–	3.0 [2.0;4.7]	–
Spain (N = 70)	–	–	3.6 [1.6;8.3]	3.5 [1.2;10.3]
Switzerland (N = 63)	–	–	3.4 [1.4;8.3]	–
UK | Netherlands | Finland (N = 344)	0.058 [0.048;0.072]	–	–	–
UK | Netherlands | Finland | Belgium (N = 448)	–	–	0.031 [0.023;0.042]	0.1 [0.013;0.77]
*Stances for using HVLA*				
HVLA-opposed (N = 1830)	Ref	Ref	Ref	Ref
Implicite-authority (N = 285)	10.1 [6.2;16.7]	5.8 [3.4;10.0]	20.3 [9.4;43.8]	8.5 [3.8;19.1]
Procedural-ethical (N = 200)	13.6 [8.1;16.7]	7.5 [4.3;13.1]	26.3 [12.0;57.7]	10.7 [4.6;24.5]
Precaution-informed (N = 292)	17.4 [11.0;27.6]	11.5 [7.0;18.9]	35.9 [17.3;74.4]	18.8 [8.7;40.9]

CI, Confidence Interval; n.s., non significant (*p* ≥ 0.05); OR, Odds Ratio; * Increased odds for every 10 years of experience, † Increase in odds for every 10% increase in proportion

### Sensitivity analysis

From the 777 respondents who considered HVLA techniques could be justified under certain conditions (permissive stances), 269 (34.6%) did not consider parental/guardian consent to be necessary. Reporting whether consent would be necessary or not was independent of whether respondents were using HVLA (R^2^ = 0.005; *p* = 0.372). There were 39 respondents who reported using HVLA techniques without considering consent as necessary. They were more likely to be older practitioners (OR per 10 years = 1.56, *p* = 0.001), be from Germany (n = 16, 41.0%, OR = 2.1, *p* = 0.032), having received practical supervision when learning HVLA techniques on newborns and infants (OR = 4.4, *p* < 0.001), and received paediatric training in a postgraduate settings (n = 17, 43.6%).

## Discussion

### Summary of findings

This study examined the use of HVLA techniques in paediatric care and their inclusion in osteopathic training programmes for infants (< 2 years), children (2–12 years), and adolescents (13–17 years). Survey results showed that 93.5% of practitioners did not use HVLA techniques in newborns and infants, and among those who did, 84.9% reported infrequent use. Approximately half reported use in children, increasing to a majority in adolescents.

Clear international variation was observed, with the highest use in newborns and infants reported in Germany, Spain, and Switzerland, and the lowest in Finland, the Netherlands, and the United Kingdom. A majority (70.2%) of respondents indicated that no condition justifies the use of HVLA techniques in paediatrics, suggesting broad disapproval of its use in paediatrics, although some clinical scenarios might arguably support its use from an evidence-based perspective where potential benefits outweigh risks and plausible mechanisms are present.

### Comparison with other studies

There is a paucity of osteopathic research available for direct comparison with these findings. Nevertheless, data from a single U.S. outpatient clinic documented osteopathic techniques used in paediatric care for 537 patients across 1,688 clinical encounters over a three-year period [30]. When similar age categories are compared, HVLA use was consistently higher in the present study: 6.5% versus 0.5% in newborns and infants, 43.5% versus 6.4% in children, and 78.3% versus 11.7% in adolescents [30]. In contrast, a survey of 245 Canadian chiropractors indicates that HVLA use is even more prevalent in chiropractic care, with 16.3% of infants, 60.0–78.8% of children, and 88.6% of adolescents receiving HVLA techniques [63]. However, given differences in methodology and study samples, direct comparison should be interpreted cautiously. Although our findings indicate that HVLA techniques are used by osteopathic practitioners to some extent in paediatric populations, their relative frequency may appear limited when considered alongside the broader range of manual techniques employed. Clinic-based data indicate that myofascial release, muscle energy, balanced ligamentous tension, and osteopathic cranial manipulative medicine are most commonly used [30, 64].

### Strengths and limitations

This is the first international professional initiative to examine the use of HVLA techniques in paediatric management by osteopathic practitioners and their inclusion in osteopathic education programmes prior to formulating professional recommendations. It was done across 24 European countries, as well as Australia, Brazil, Israel, and New Zealand. The survey was available in 11 languages and underwent validation through two pilot studies. It was distributed to members of recognised professional associations that require minimum standards regarding training, insurance, continuing professional development, and adherence to a code of ethics.

The findings of this study should also be interpreted in light of its limitations. The survey was distributed in 28 countries, although 13 countries were the main contributors (i.e., > 50 responses), representing a substantial regional sample of the 46 countries in which osteopathy is practised worldwide [29]. Most responding and highly responding countries were European, and response rates varied considerably (0.0–53.7%), limiting the generalisability of the findings. Future surveys may consider extending the recruitment period or offering incentives to increase response rates. The invited population (n = 24,261) and respondents (n = 2607) represent approximately 12.3% and 1.3%, respectively, of the global osteopathic workforce (or 30.6% and 3.3% respectively, excluding U.S.-based osteopathic physicians) [65]. Although this suggests a degree of international representation, the recruitment approach limits generalisability and introduces potential selection bias by excluding non-members of participating associations (coverage bias). In addition, the survey may have preferentially attracted practitioners involved in, or interested in, paediatric care (self-selection bias), as some invitees initially perceived the study as targeting paediatric practice, which is reflected in the relatively high proportion of paediatric patients reported by respondents. Despite revisions to the invitation materials, which emphasised that all osteopaths were invited, recruitment bias cannot be ruled out. Furthermore, many countries were not represented in the surveys; most notably, the U.S. was not included, although it accounts for a large share of the global osteopathic workforce. Regardless of differences in scope of practice between countries [29], it seems important to cover all countries that have implemented osteopathy in their healthcare system. Also, due to the collection of anonymised data, duplicate responses could not be identified in this survey, which limits the generalisability of the findings because independent individual responses cannot be assured. Similarly, to protect participant anonymity, the surveys did not collect data on the countries where osteopathic practitioners received their education and OEIs delivered their osteopathic training, which precludes further discussion about potential educational and regional determinants of paediatric HVLA use.

Finally, certain statutory scope-of-practice restrictions must be acknowledged, because osteopathic practitioners are not legally permitted to treat children under 8 years in Sweden [66], manipulate the spine of infants under 6 months in France [67], and perform HVLA techniques in any paediatric or adult patient in Canada [68]. The professional osteopathic associations from these three countries (i.e., member organisations of OE) consequently expressed some concerns regarding the participation of their members in the survey. While the Swedish and French associations agreed to distribute the survey as a means of quality control and adherence to existing recommendations, the Canadian association deemed it inappropriate to distribute the survey, which led to the under-representation of Canadian osteopaths within the survey.

### Practical implications

A notable proportion of respondents considered patient consent sufficient to justify conditional HVLA use in paediatrics, despite the fact that legally valid consent is typically provided by parents or guardians. This suggests potential misunderstandings of informed consent—possibly interpreted as non-verbal or “tissue-based” assent rather than formal legal consent—and highlights the need to reinforce professional and legal obligations. Reassuringly, HVLA use was most strongly associated with a precaution-informed stance, specific training, and practical supervision. Education programs in paediatrics would gain in explicitly including consent procedures in their teaching objectives and evaluations.

The absence of HVLA techniques from some osteopathic training curricula may partly be attributable to the limited quality of evidence regarding their effectiveness and the resulting uncertainty about their role in reducing paediatric symptoms [69]. Public and regulatory concerns may also be reflected in decisions about including HVLA techniques in undergraduate and postgraduate education, particularly regarding newborns and infants. In Australia, a governmental safety review on paediatric HVLA use has outlined educational recommendations [46], and the interim policy by a professional regulatory board prohibiting paediatric HVLA use [48]. Both are likely to shape current and future educational frameworks, as seen in naturopathy, where educators have reported adapting their educational curricula to changes in regulation and recommendations [70]. Educational provision appears more common for adolescents; however, it remains unclear whether such training is specifically adapted to the anatomical, physiological, and developmental characteristics of this age group or largely derived from adult-oriented teaching models. Educational standards for osteopathic paediatric care should specify that reflective structured supervised clinical training with infants, children and adolescents are indispensable to validate any form of training.

Our findings should also be interpreted within the current scientific and policy context, in which several professional and regulatory bodies have recommended extreme caution or even prohibition of spinal manipulation in paediatric populations [45–49], with some arguing it is “time to stop the madness” [52]. Such positions are largely based on limited evidence of effectiveness [39, 40] and ongoing concerns regarding safety [39–44], including the potential for serious adverse events [46]. The results of the present surveys reveal that osteopaths apply HVLA techniques in infants, children, and adolescents, yet there is a relatively limited number of serious adverse events described in the literature [39–44]. Although the present study did not assess adverse events directly, this discrepancy may reflect either an underreporting of harms [39–41] or a possible overestimation of risks (due to methodological limitations) [43] associated with paediatric spinal manipulation. Existing literature generally suggests underreporting of harms [39–41], but overestimation cannot be excluded due to methodological limitations. Much of the evidence is based on case reports and observational studies, which are useful for identifying rare adverse events but are not suitable for establishing causality or estimating risk [43]. In contrast, longitudinal studies have predominantly reported mild to moderate adverse events, with no serious events identified [39, 40, 43, 44, 71]. This is consistent with longitudinal evidence in adults, where cervical HVLA showed no increased risk of adverse events compared with control interventions in a meta-analysis of randomised controlled trials [72]. Moreover, several serious adverse events reported in the literature may be attributable to pre-existing but unrecognised pathology [40, 42]. Nevertheless, any serious adverse events in paediatric patients—such as rib fractures, temporary paralysis, or death—are unacceptable, particularly when alternative safe and effective treatment options are available, and must therefore be considered with utmost caution [45, 71]. A precautionary approach to paediatric HVLA techniques thus appears justified based on current evidence and the foundational bioethical principle of *first do no harm*. Furthermore, this approach is operationalised by integrating patient preferences, clinician expertise, and a robust informed consent process—collectively reflecting the core tenets of evidence-based practice.

Professional regulation did not seem to be associated with the observed international differences, as both high- and low-utilisation groups include countries in which osteopathy is formally regulated (Finland, France, UK, and Switzerland). Notably, in France, despite regulatory status and specific scope-of-practice restrictions concerning cervical HVLA use in infants, 3.4% of respondents indicated that they used this technique. Guidelines, on the other hand, may show a stronger correlation with practice behaviors, as the Netherlands has issued national clinical guidelines prohibiting HVLA use in children aged 0–2 years (0%) [55], along with additional recommendations to avoid HVLA techniques in the upper cervical region [73]. Policy makers might benefit from considering that managing safety in osteopathic care could be better achieved through professional guidance rather than external legal regulation that is not fully supported by the profession.

Further research is required to inform guidelines and regulatory decisions aimed at safeguarding infants, children, and adolescents [45]. We therefore call for comprehensive investigation of harms associated with paediatric HVLA techniques. This should include systematic monitoring of adverse events—through mandatory or voluntary reporting mechanisms involving healthcare institutions, professional associations, insurers, or regulatory authorities, depending on national frameworks—as well as targeted research efforts. Previous research showed that active, instead of passive, surveillance should be preferred [74] and a specific, valid, and feasible online reporting system for monitoring AEs after HVLA in paediatric patients has already been developed [75, 76]. Research efforts should prioritise longitudinal and observational studies examining HVLA-related harms in paediatrics and improved, guideline-conform reporting of adverse events in manual therapy research more broadly. Future research should also explore osteopathic practitioners’ beliefs and attitudes toward HVLA use in paediatrics—particularly the reasons underlying the general opposition—using qualitative methods such as interviews.

## Conclusion

Survey results indicate that 19 out of 20 osteopaths never use HVLA techniques in newborns and infants, and those who do report infrequent use. Utilisation increases with age, with about half reporting use in children and a majority in adolescents. Most respondents consider HVLA techniques unjustified in paediatrics, although respondents with more permissive stances reported greater use, often alongside specific training, while those with a precaution-informed stance showed the strongest association with paediatric practice.

Given that a non-negligible proportion of practitioners reported using HVLA techniques in paediatric populations, the development of an evidence-based professional position statement for the osteopathic profession is warranted, alongside robust research to clarify the safety and effectiveness of these techniques in paediatrics to inform ethically and clinically sound practice.

## Supplementary Information

Additional file1 (DOCX 27 KB)

Additional file2 (DOCX 2663 KB)

## Data Availability

The datasets supporting the conclusions of this article are available on Zenodo, [https://zenodo.org/records/19097090]. Details on commands and full output file are also made available on Zenodo (4–5).

## Abbreviations

AE: Adverse events
AIC: Akaike information criterion
aOR: Adjusted odds ratio
BIC: Bayesian Information Criterion
CI: Confidence Interval
CPD: Continuing Professional Development
HVLA: High velocity, low amplitude
LCMM: Latent class marginal means
OE: Osteopathy Europe
OEI: Osteopathic Education Institute
OR: Odds ratio
PIS: Participant information sheet
R^2^: Coefficient of determination
SD: Standard deviation
WHO: World Health Organisation
